# RODAN: a fully convolutional architecture for basecalling nanopore RNA sequencing data

**DOI:** 10.1186/s12859-022-04686-y

**Published:** 2022-04-20

**Authors:** Don Neumann, Anireddy S. N. Reddy, Asa Ben-Hur

**Affiliations:** 1 Department of Computer Science, Colorado State University, 1873 Campus Delivery, Fort Collins, CO 80523-1873 USA; 2 Department of Biology, Colorado State University, 1878 Campus Delivery, Fort Collins, CO 80523-1878 USA

**Keywords:** RNA basecalling, Oxford nanopore, Long read sequencing, Convolutional networks

## Abstract

**Background:**

Despite recent progress in basecalling of Oxford nanopore DNA sequencing data, its wide adoption is still being hampered by its relatively low accuracy compared to short read technologies. Furthermore, very little of the recent research was focused on basecalling of RNA data, which has different characteristics than its DNA counterpart.

**Results:**

We fill this gap by benchmarking a fully convolutional deep learning basecalling architecture with improved performance compared to Oxford nanopore’s RNA basecallers.

**Availability:**

The source code for our basecaller is available at: https://github.com/biodlab/RODAN.

**Supplementary Information:**

The online version contains supplementary material available at 10.1186/s12859-022-04686-y.

## Introduction

Oxford nanopore sequencing presents an opportunity for advancements in genomics, transcriptomics, and epitranscriptomics because of its ability to directly sequence a DNA or RNA strand without requiring amplification, producing long reads that can help identify splice isofroms unambiguously, determine poly(A) length, and can potentially capture information on base modifications [1, 2]. Sequencing of both DNA and RNA using this technology occurs by passing nucleotide strands through a synthetic protein pore that straddles a membrane, and recording the resulting current across the membrane. The technology has been developing at a rapid pace since its release in 2014 based on its capabilities for generating long DNA reads and the more recent application to direct RNA sequencing [2]. However, while the technology offers many advantages over other long and short read technologies, it is unfortunately hampered by high error rates [1].

Decoding the current generated by a nucleotide strand as it passes through the pore is a challenging task. This is due to several factors [1]. First, the signal associated with each nucleotide passing through the pore is affected by its surrounding nucleotides (typically, two on each side). Second, the speed of a strand translocating through the pore varies. Therefore, the resulting signal produced by a polymer is a one dimensional sequence of real numbers where each nucleotide is represented by a variable number of sequence values. We will denote this as the *samples per base* associated with a nucleotide. And finally, the electrical signal measured in picoamps, is very noisy. All these factors makes basecalling highly error prone [1, 3].

Improving basecalling accuracy has been the focus of several recent research papers. The earliest approaches were based on recurrent neural networks such as Chiron [4], DeepNano [5] and Oxford Nanopore Technologies’ (ONT) Albacore, which was replaced with Guppy. However, the current trend has been towards fully convolutional architectures such as in ONT’s development basecaller Bonito [6] which is based on Nvidia’s speech recognition network Quartznet [7]. Further attempts have been made utilizing attention mechanisms as in SACall [8]. While Bonito has improved DNA basecalling accuracy slightly, there is still much room for improvement before ONT’s technology can match the accuracy of Illumina sequencing. These improvements are likely to come in both basecalling and the technology itself. Despite all this recent research, most of it focused on DNA data, with little attention to basecalling of RNA data. RNA is sampled by the pore at 70 bases per second (bps), compared to 450 bps for DNA, leading to different signal characteristics, and requiring basecalling methods specifically trained for this data. In this paper we introduce RODAN: **R**NA nan**O**pore **D**ecoding with convolution**A**l **N**etworks, a fully convolutional architecture that achieves state-of-the-art performance on transcriptome data from multiple species including animals and plants.

## Methods

### Architecture and training

**Fig. 1:**
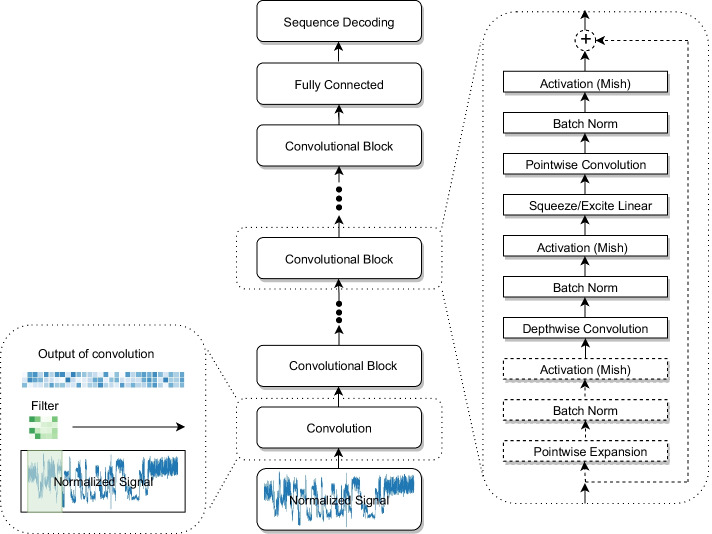
The RODAN architecture. The normalized signal is passed through a succession of convolutional blocks which gradually incorporate surrounding information. Each block is composed of several processing steps (convolution, activation, batch normalization etc.), which are standard building blocks in the construction of deep neural networks. The final output is passed through a fully connected layer to produce the decoded sequence of nucleotides

We propose a fully convolutional basecalling neural network which takes an intuitive approach to decoding the signal generated by ONT direct RNA sequencing. Convolutional networks have emerged as an important technique for working with noisy one dimensional signals [9], and are therefore a good approach for decoding the signal generated by ONT data. A convolutional network scans the input signal with a set of filters or kernels that make up its convolutional layer (see Fig. 1). Each of these kernels computes a function over a small segment of the signal; the results of the local computation are then fed to the next layer of computation, and can be stacked to create multi-layer “deep” models.

There is much recent research on the design of deep convolutional networks, and the architecture for RODAN is inspired by Google’s EfficientNet [10]. EfficientNet improved the state of the art in image classification while simultaneously reducing the number of model parameters by an order of magnitude. The RODAN architecture is composed of 22 convolutional blocks, contains roughly 10 million parameters, and utilizes a similar convolutional block structure (see Fig. 1). RODAN gradually incorporates surrounding information for each position in the signal by increasing the kernel size with each successive convolutional block. By increasing the kernel size, we expand the window size to incorporate surrounding signal information which accommodates for the variable samples per base and gathers the necessary information from neighboring nucleotides for accurate decoding. We note that the training and validation set were sampled from data where roughly 83% of all chunks of 4096 values ranged between 20 and 70 samples per base.

#### Architecture details

The RODAN architecture is composed of 22 convolutional blocks and contains around 10M parameters. The first block is a regular convolution with a kernel size of 3 which acts as a “smoothing” layer to denoise the signal. The smoothing convolution is followed by a squeeze and excitation block. The remaining blocks, as depicted in Fig. 1, are composed of separable convolutions, where depthwise convolution is followed by a squeeze and excitation block, then a pointwise convolution [11]. All squeeze and excitation blocks forego using a reduction ratio and instead reduce to a fixed size of 32. When the number of channels increases between layers, the convolutional block also includes a pointwise expansion to increase the number of channels before the depthwise convolution. Each convolution operation is followed by a batchnorm and is passed through the Mish activation function [12]. The Mish activation function also replaces ReLU in the squeeze and excitation block. In addition, residual connections have been replaced with ReZero [13] which parameterizes the residual addition.

In our architecture, we increase the number of channels and the kernel sizes used in each layer, up to 768 channels and a kernel size of 100 in the final layer. Its output is then fed to a fully connected layer, followed by a classification layer with a log softmax activation function. The connectionist temporal classification (CTC) loss [14] was used as the objective function for training the network. We use the Ranger optimizer version 0.1.1 [15], which combines RAdam [16] with lookahead [17], with an initial learning rate of 0.002 and the default weight decay of 0.01. Learning rate decay is utilized at a rate of 0.5 with a scheduler patience of 1 and a threshold of 0.1. Only 1000 batches were used for validation. The neural network architecture is detailed in Additional file 1 Table S1.

#### Model training

Training of RODAN was performed on an HP Z440 workstation with 6x3.6Ghz dual core processors, 16 GB of RAM, and an Nvidia Titan V GPU with 12 GB of memory. Training was performed using PyTorch version 1.5.1 with the maximum possible batch size of 30 and stopped after 20 epochs. Label smoothing was also utilized by reweighting the blanks in the CTC sequence with a higher probability of 0.1. The nucleotide vocabulary is then reweighted uniformly at 0.025. Basecalling is performed with a beam search size of 5.

Training of Taiyaki was performed utilizing version 5.0.0 on the same hardware setup. We used the suggested RNA training parameters which are a base layer size of 256, a stride of 10, and number of epochs equal to 10.

### Data

*Training data.* The RNA training data was selected from samples from an in house *Arabidopsis thaliana* wild type which utilized flow cell version R9.4.1, Epinano synthetic constructs (R9.4.1) which contain all possible 5-mers ([18], *Homo Sapiens* (R9.4) from the NA12878 project (BHAM_Run1) [19], *Caenorhabditis elegans* (R9.4) from [20], and *Escherichia coli* (R9.4) from [21].

To generate the Arabidopsis data, total RNA from 17 days-old Arabidopsis thaliana Col-0 seedlings grown on $\frac{1}{2}$ MS at $20^{\circ }C$ (16/8 hrs light/dark cycle) was isolated using TRIzol reagent and suspended in $160 \mu l$ of DEPC-treated water. DNAse treatment was performed by adding $20 \mu l$ of 10x DNase buffer and $20 \mu l$ RNAse-free DNAseI and incubated for 30 minutes at $37^{\circ }C$. RNA was then purified using phenol/chloroform. Poly(A)+ mRNA was isolated from about $150 \mu$g of total RNA using the Oligotex Direct mRNA kit (Qiagen). One $\mu g$ of poly(A)+ RNAs was converted into a library with the Direct RNA Library kit SQK-RNA002 (Oxford nanopore). The library was sequenced on a SpotON R9.4.1 FLO-MIN106 flowcell, using a GridION x5 sequencer.

All reads were first basecalled with Guppy version 3.4.5 followed by a Tombo version 1.5.1 [22] resquiggle to assess the alignment qualities using the signal matching score and qscore provided by Tombo. The signal matching score (SMS) assesses the quality of the raw current signal against the expected signal, where higher scores indicate lower quality. As Tombo uses a default of 2 for RNA, all reads were filtered with $\le 2$ for the SMS. All reads were filtered with $\ge 11$ for qscore, except for the *E. coli* sample which used $\ge 8$ for the qscore due to the low quality of the reads. The remaining reads for each sample were then processed using Oxford nanopore’s research training model Taiyaki according to their instructions [23]. Taiyaki stores the resulting data in an HDF5 file which includes the raw signal data for each read, along with its genomic sequence and alignment positions.

The resulting HDF5 file is comprised of 116,072 reads, 24,370 from Arabidopsis aligned to the Araport11 [24] transcriptome, 29,728 from Epinano synthetic constructs aligned to their released reference [18], 30,048 from *H. Sapiens* (BHAM_Run1) aligned to v33 of the gencode [25] transcriptome, 24,192 from *C. elegans* aligned to the CE11 [20] transcriptome, and 7734 from *E. coli* aligned to the transcriptome generated from the genome and annotations in the NCBI assembly database [26]. Both the Arabidopsis and *E. Coli* transcriptomes were generated from their respective genomes and gff annotations using the gffread command from cufflinks [27] with the -O option to add non-transcript records.

From this dataset, reads were randomly selected for either training or validation purposes. Each read had a random starting point chosen between 0 and 1024 signal values, and was then segmented into chunks of 4096 values where only chunks with a maximum of 15 samples per base were selected. After a million chunks are selected for training, the remaining reads are then used to select 100, 000 chunks for validation. The raw input signals are normalized by median absolute deviation.

*Test data.* The test set for measuring the accuracy of our basecaller is comprised of five different samples. These samples originated from studies distinct from those used to generate our training data except for the human data which is taken from a different lab from the nanopore WGS Consortium’s NA12878 project [19]. For each sample, a selection of reads was basecalled with Guppy v4.4.0. Any read which aligned to the mitochondrial genome was discarded. Of the basecalled reads which aligned to each transcriptome, 100,000 were randomly selected for inclusion in the dataset.

The RNA test data is composed from datasets originating from multiple species. Data for *Homo sapiens* (R9.4) was selected from the NA12878 project (BHAM_Run1) [19] and aligned to v36 of the gencode human transcriptome [25]. *Arabidopsis thaliana* (R9.4) data is the Col-0 wildtype from [28] aligned to the Araport11 [24] transcriptome. *Mus musculus* (R9.4.1) is from [29] and aligned to the vM25 gencode transcriptome. *S. cerevisiae S288C* (R9.4.1) from [30] is aligned to the transcriptome from the NIH genome database [31]. The *Populus trichocarpa* (R9.4.1) from [32] is aligned to the transcriptome generated from the genome and annotations in the NIH assembly database [33]. The Poplar transcriptome, in addition to the Arabidopsis, were generated in the same manner as the transcriptomes for the training data.

### Evaluation

Basecallers were evaluated using sequence identity is defined as:

1 $$\begin{aligned} \textit{accuracy} = \frac{M}{M + S + I + D} \end{aligned}$$

where *M* is the number of matching bases, *S* is the number of mismatches, *I* is the number of insertions, and *D* is the number of deletions. Two sample t-tests were performed using the scipy stats function ttest_ind comparing the results between RODAN and Guppy, and RODAN and Taiyaki.

## Results and discussion

We compared RODAN to other available RNA basecallers which include the latest release of ONT’s production basecaller Guppy and their research software Taiyaki [34]. Taiyaki was trained with our generated training data. Both Guppy and Taiyaki are based on recurrent neural networks (RNNs). The inherently sequential processing of data with RNNs interferes with modeling long term dependencies and parallelization. Convolutional architectures on the other hand are easily parallelizable.

**Fig. 2:**
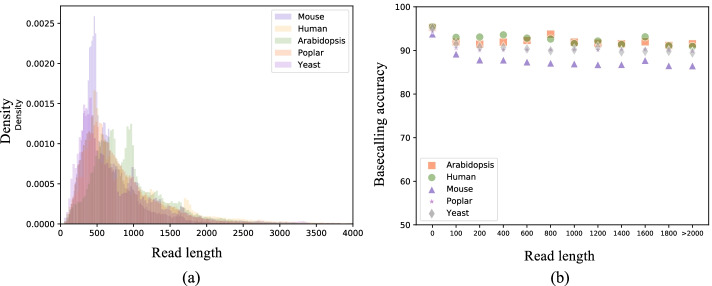
Read statistics. For each of the five datasets we show histograms of read length in (**a**), and basecalling calling accuracy as a function of read length

For our evaluation we used the five benchmark datasets described above to assess the accuracy of RODAN across multiple species. Read length distributions across datasets were very similar as seen in Fig. 2a. Basecalled reads were aligned with minimap2 2.17-r941 [35] against the respective transcriptomes [detailed in Methods]. All supplementary alignments were discarded. Basecalling accuracy is shown in Table 1, reported as median sequence identity, similarly to other papers reporting basecalling accuracy [3]. We observe that RODAN outperforms Guppy and Taiyaki in all five datasets, with the largest difference in human. The only exception is in yeast, where Guppy was able to match RODAN’s performance. All the differences except for RODAN vs Guppy in yeast were highly statistically significant (p-value less than $2.75 10^{-157}$ using a t-test applied as described in Methods). We note that all three basecallers had difficulty with the mouse dataset. This may be the result of not having trained the model on mouse data. To test this hypothesis, we added 24,295 reads of mouse data from [30] to the training set and retrained RODAN with the same configuration. This increased the median accuracy of the mouse from $87.99\%$ to $89.37\%$. However, it decreased human median accuracy by $1.2\%$ and increased the number of unaligned reads across the remainder of the test data. In poplar, another eukaryote, the model performs well despite not having been trained on data from it. We also report on the total amount of unaligned reads for each basecaller. Taiyaki, which was trained on our generated training dataset, performed slightly better in that regard. We note that the dataset was prepared with Guppy, hence the number of unaligned reads is not applicable.

**Table 1:** Basecalling accuracy computed using percent identity and number of unaligned reads across datasets for Guppy 4.4.0, Taiyaki 5.0, and RODAN 1.0

Dataset	Basecaller	Median Accuracy	Unaligned
Human [19]	Guppy	90.60	N/A
	Taiyaki	91.16	900
	RODAN	**93.23**	1307
Mouse [29]	Guppy	87.65	N/A
	Taiyaki	86.25	3079
	RODAN	**87.99**	2819
Arabidopsis [28]	Guppy	91.59	N/A
	Taiyaki	91.10	957
	RODAN	**92.89**	1001
Poplar [32]	Guppy	90.16	N/A
	Taiyaki	89.72	1598
	RODAN	**91.11**	1652
Yeast [30]	Guppy	91.35	N/A
	Taiyaki	90.01	2721
	RODAN	**91.41**	3035

For each dataset, the best result is shown in bold

Each dataset contains 100, 000 reads. Only reads alignable by Guppy were used to build each dataset, hence the N/A for unaligned reads. Additional basecalling statistics are provided in Additional file 1: Table S2

To obtain more detailed understanding of model performance, we show basecalling accuracy as a function of read length in Fig. 2b and Supplementary Fig. 1. In all datasets we observe a slight decrease in accuracy with read length. We hypothesize that shorter length reads have less of a tendency to form structures that would impede their movement though the pore, leading to more accurate basecalls. In our experiments, Guppy ran around 7x faster than RODAN. This is to be expected since Guppy is optimized production code that is written in C++. Both were run on an HP Z440 workstation with 6x3.6Ghz dual core processors, 16 GB of RAM, and an Nvidia Titan V GPU with 12 GB of memory.

## Conclusions

We presented RODAN, an RNA basecaller for ONT data with state-of-the-art accuracy. Our approach accounts for the varied samples per base and high level of noise inherent to this data with a convolutional architecture that gradually incorporates surrounding information to correctly decode each nucleotide. The software is freely available, and can form the basis for further development. In addition, we have also assembled and released the first comprehensive dataset that can be used to test the accuracy of RNA basecallers in future research [36]. To our chagrin, many published studies do not release raw ONT fast5 data which is crucial to method development and re-analysis of data. We hope this trend improves in the future.

## Supplementary Information

**Additional file 1.** Supplementary tables and figures, including details of the RODAN architecture and additional performance results.

## Data Availability

The datasets supporting the conclusions of this article are available from the following sources: Raw RNA training data was obtained from: NA12878 RNA data from the nanopore WGS Consortium (BHAM_RUN1) [37]; Epinano synthetic construct data is from SRA accession SRX5177013; *C. elegans* data is from ENA accession ERX3290223; *E. coli* data is from SRA accession SRX8495663; Arabidopsis data is from SRA accession PRJNA803210. Taiyaki RNA HDF5 data: DOI https://doi.org/10.5281/zenodo.4556884 [38]. Processed RNA training and validation dataset: DOI https://doi.org/10.5281/zenodo.4556950 [39]. RNA test data sourced from: NA12878 RNA data from nanopore WGS Consortium (HOPKINS_RUN1) [37]; Arabidopsis data is from ENA accession ERX3766448; mouse data is from ENA accession ERX3444723; poplar data is from SRA accession ERX3444723; yeast data is from SRA accession SRX8120036 [40]. RNA test dataset: DOI https://doi.org/10.5281/zenodo.4557004 [36]. Software availability: RODAN is available under the MIT license from https://github.com/biodlab/RODAN

## References

[1] Amarasinghe SL, Su S, Dong X, Zappia L, Ritchie ME, Gouil Q (2020). Opportunities and challenges in long-read sequencing data analysis. Genome Biol.

[2] Garalde DR, Snell EA, Jachimowicz D, Sipos B, Lloyd JH, Bruce M, Pantic N, Admassu T, James P, Warland A, Jordan M, Ciccone J, Serra S, Keenan J, Martin S, McNeill LE, Wallace J, Jayasinghe L, Wright C, Blasco J, Young S, Brocklebank D, Juul S, Clarke J, Turner DJ (2018). Highly parallel direct RNA sequencing on an array of nanopores. Nat Methods..

[3] Wick RR, Judd LM, Holt KE (2019). Performance of neural network basecalling tools for oxford nanopore sequencing. Genome Biol.

[4] Teng H, Cao MD, Hall MB, Duarte T, Wang S, Coin LJ (2018). Chiron: translating nanopore raw signal directly into nucleotide sequence using deep learning. GigaScience.

[5] Boža V, Brejová B, Vinař T (2017). DeepNano: deep recurrent neural networks for base calling in minion nanopore reads. PloS One.

[6] Bonito Basecaller. http://github.com/nanoporetech/bonito. Accessed 21 Feb 2021.

[7] Kriman S, Beliaev S, Ginsburg B, Huang J, Kuchaiev O, Lavrukhin V, Leary R, Li J, Zhang Y. Quartznet: Deep automatic speech recognition with 1d time-channel separable convolutions. In: ICASSP 2020-2020 IEEE international conference on acoustics, speech and signal processing (ICASSP). IEEE; 2020. pp. 6124–6128.

[8] Huang N, Nie F, Ni P, Luo F, Wang J. SACall: a neural network basecaller for oxford nanopore sequencing data based on self-attention mechanism. IEEE/ACM Trans Comput Biol Bioinform. 2020. 10.1109/TCBB.2020.3039244 33211664

[9] Fawaz HI, Forestier G, Weber J, Idoumghar L, Muller P-A (2019). Deep learning for time series classification: a review. Data Min Knowl Discov.

[10] Tan M, Le Q. EfficientNet: rethinking model scaling for convolutional neural networks. In: International conference on machine learning; 2019. PMLR. pp. 6105–6114.

[11] Chollet F. Xception: deep learning with depthwise separable convolutions. In: Proceedings of the IEEE conference on computer vision and pattern recognition; 2017. pp. 1251–1258.

[12] Misra D. Mish: a self regularized non-monotonic activation function; 2019. arXiv preprint arXiv:1908.08681.

[13] Bachlechner T, Majumder BP, Mao HH, Cottrell GW, McAuley J. Rezero is all you need: fast convergence at large depth; 2020. arXiv preprint arXiv:2003.04887.

[14] Graves A, Fernández S, Gomez F, Schmidhuber J. Connectionist temporal classification: labelling unsegmented sequence data with recurrent neural networks. In: Proceedings of the 23rd international conference on machine learning; 2006. pp. 369–376.

[15] Ranger Optimizer. http://github.com/mpariente/Ranger-Deep-Learning-Optimizer. Accessed 21 Feb 2021.

[16] Liu L, Jiang H, He P, Chen W, Liu X, Gao J, Han J. On the variance of the adaptive learning rate and beyond; 2019. arXiv preprint arXiv:1908.03265.

[17] Zhang MR, Lucas J, Hinton G, Ba J. Lookahead optimizer: k steps forward, 1 step back; 2019. arXiv preprint arXiv:1907.08610.

[18] Liu H, Begik O, Lucas MC, Ramirez JM, Mason CE, Wiener D, Schwartz S, Mattick JS, Smith MA, Novoa EM (2019). Accurate detection of m6a RNA modifications in native RNA sequences. Nat Commun.

[19] Workman RE, Tang AD, Tang PS, Jain M, Tyson JR, Razaghi R, Zuzarte PC, Gilpatrick T, Payne A, Quick J (2019). Nanopore native RNA sequencing of a human poly(A) transcriptome. Nat Methods.

[20] Roach NP, Sadowski N, Alessi AF, Timp W, Taylor J, Kim JK (2020). The full-length transcriptome of *C. elegans* using direct RNA sequencing.. Genome Res.

[21] Grünberger F, Knüppel R, Jüttner M, Fenk M, Borst A, Reichelt R, Hausner W, Soppa J, Ferreira-Cerca S, Grohmann D. Exploring prokaryotic transcription, operon structures, rRNA maturation and modifications using nanopore-based native RNA sequencing. bioRxiv, 2020:2019–12.

[22] Tombo. http://github.com/nanoporetech/tombo. Accessed 21 Feb 2021.

[23] Taiyaki walk-through. http://github.com/nanoporetech/taiyaki/blob/master/docs/walkthrough.rst. Accessed 21 Feb 2021.

[24] Cheng C-Y, Krishnakumar V, Chan AP, Thibaud-Nissen F, Schobel S, Town CD (2017). Araport11: a complete reannotation of the arabidopsis thaliana reference genome. Plant J.

[25] Frankish A, Diekhans M, Ferreira A-M, Johnson R, Jungreis I, Loveland J, Mudge JM, Sisu C, Wright J, Armstrong J (2019). Gencode reference annotation for the human and mouse genomes. Nucleic Acids Res.

[26] ASM584v2-Genome-Assembly-NCBI. https://www.ncbi.nlm.nih.gov/assembly/GCF_000005845.2. Accessed 21 Feb 2021.

[27] Trapnell C, Williams BA, Pertea G, Mortazavi A, Kwan G, van Baren MJ, Salzberg SL, Wold BJ, Pachter L (2010). Transcript assembly and abundance estimation from RNA-seq reveals thousands of new transcripts and switching among isoforms. Nat Biotechnol.

[28] Parker MT, Knop K, Sherwood AV, Schurch NJ, Mackinnon K, Gould PD, Hall AJ, Barton GJ, Simpson GG (2020). Nanopore direct RNA sequencing maps the complexity of arabidopsis mRNA processing and ma modification. Elife.

[29] Bilska A, Kusio-Kobiałka M, Krawczyk PS, Gewartowska O, Tarkowski B, Kobyłecki K, Gruchota J, Borsuk E, Dziembowski A, Mroczek S. B cell humoral response and differentiation is regulated by the non-canonical poly (a) polymerase tent5c. bioRxiv, 2019:686683. 10.1038/s41467-020-15835-3 PMC7184606 32341344

[30] Jenjaroenpun P, Wongsurawat T, Wadley TD, Wassenaar TM, Liu J, Dai Q, Wanchai V, Akel NS, Jamshidi-Parsian A, Franco AT (2021). Decoding the epitranscriptional landscape from native RNA sequences. Nucleic Acids Res.

[31] Saccharomyces cerevisiae S288C (ID 15)-Genome-NCBI. https://www.ncbi.nlm.nih.gov/genome/15?genome_assembly_id=22535. Accessed 21 Feb 2021.

[32] Gao Y, Liu X, Wu B, Wang H, Xi F, Kohnen MV, Reddy AS, Gu L (2021). Quantitative profiling of n 6-methyladenosine at single-base resolution in stem-differentiating xylem of populus trichocarpa using nanopore direct RNA sequencing. Genome Biol.

[33] Pop_tri_v3-Genome-Assembly - NCBI. https://www.ncbi.nlm.nih.gov/assembly/GCF_000002775.4/. Accessed 21 Feb 2021.

[34] Taiyaki research software. http://github.com/nanoporetech/taiyaki. Accessed 21 Feb 2021.

[35] Li H (2018). Minimap2: pairwise alignment for nucleotide sequences. Bioinformatics.

[36] Neumann D, Reddy ASN, Ben-Hur A. Oxford nanopore RNA test dataset for RODAN. 10.5281/zenodo.4557004. Accessed 1 Apr 2021.

[37] NA12878 RNA data. http://github.com/nanopore-wgs-consortium/NA12878/blob/master/RNA.md. Accessed 21 Feb 2021.

[38] Neumann D, Reddy ASN, Ben-Hur A. Oxford Nanopore RNA training and validation data for RODAN. 10.5281/zenodo.4556884. Accessed 1 Apr 2021.

[39] Neumann D, Reddy ASN, Ben-Hur A. Oxford nanopore RNA training and validation data for RODAN. 10.5281/zenodo.4556950. Accessed 1 Apr 2021.

[40] SC_YPD_heatshock_ctrl_fast5.tar.gz. https://trace.ncbi.nlm.nih.gov/Traces/sra/sra.cgi?study=SRP166020. Accessed 21 Feb 2021.

