# Supplementary material for: RODAN: a fully convolutional architecture for basecalling nanopore RNA sequencing data
Source: BMC Bioinformatics. 2022 Apr 20;23:142. doi: 10.1186/s12859-022-04686-y (PMC9020074; doi:10.1186/s12859-022-04686-y)
Supplement: Supplementary file 1 — Additional file 1. Supplementary tables and figures, including details of the RODAN architecture and additional performance results. [file 12859_2022_4686_MOESM1_ESM.pdf]

Supplementary material for: RODAN: a fully convolutional architecture for basecalling nanopore RNA sequencing data

Don Neumann, Anireddy S.N. Reddy, Asa Ben-Hur

February 28, 2022

Alignment generation

minimap2 was run with options `--secondary=no -ax map-ont --cs`. The CS tag was used to determine the mismatches, insertions, and deletions to calculate the final accuracies. The accuracy.py script is available in the project's github repository.

Details of the RODAN architecture

Block	Operator	Kernel / Stride	#Channels
1	Convolution, SQEX	3	256
2	ConvBlock	10	256
3	ConvBlock	10 / 10	256
4	ConvBlock	10	320
5	ConvBlock	15	384
6	ConvBlock	20	448
7	ConvBlock	25	512
8	ConvBlock	30	512
9	ConvBlock	35	512
10	ConvBlock	40	512
11	ConvBlock	45	512
12	ConvBlock	50	512
13	ConvBlock	55	768
14	ConvBlock	60	768
15	ConvBlock	65	768
16	ConvBlock	70	768
17	ConvBlock	75	768
18	ConvBlock	80	768
19	ConvBlock	85	768
20	ConvBlock	90	768
21	ConvBlock	95	768
22	ConvBlock	100	768

Supplementary Table 1: The RODAN network architecture. Kernel denotes the convolution kernel size and stride denotes the kernel step which defaults to 1 unless noted. #Channels denotes the number of kernels utilized. The first block is a normal convolution followed by batchnorm, activation, and a squeeze and excitation layer.

Dataset	Basecaller	Median	Average	Unaligned reads	Mis %	Del %	Ins %
Arabidopsis	Guppy	91.59	90.72	N/A	2.32	4.77	2.18
	Taiyaki	91.10	90.37	957	2.28	5.34	2.01
	RODAN	**92.89**	**92.24**	1001	1.90	3.75	2.12
	RODAN (nobeam)	92.51	91.99	1055	1.79	4.76	1.46
Mouse	Guppy	87.65	87.17	N/A	3.88	6.29	2.66
	Taiyaki	86.25	85.97	3079	4.24	7.33	2.46
	RODAN	**87.99**	**87.60**	2819	3.78	6.43	2.19
	RODAN (nobeam)	87.54	87.17	3291	3.61	7.47	1.76
Human	Guppy	90.60	89.87	N/A	2.56	5.35	2.22
	Taiyaki	91.16	90.61	900	2.19	5.27	1.94
	RODAN	**93.23**	**92.62**	1307	1.73	3.52	2.13
	RODAN (nobeam)	92.92	92.45	1086	1.62	4.59	1.34
Yeast	Guppy	91.35	**90.51**	N/A	2.78	4.20	2.51
	Taiyaki	90.01	89.29	2721	3.21	4.71	2.79
	RODAN	**91.41**	90.46	3035	2.91	4.13	2.51
	RODAN (nobeam)	91.11	90.22	3182	2.77	5.21	1.80
Poplar	Guppy	90.16	89.26	N/A	2.95	4.92	2.87
	Taiyaki	89.72	88.90	1598	3.01	5.15	2.94
	RODAN	**91.11**	**90.13**	1652	2.77	4.26	2.84
	RODAN (nobeam)	90.75	89.81	1813	2.64	5.50	2.05

Supplementary Table 2: Detailed basecalling accuracy across datasets for Guppy 4.4.0, Taiyaki 5.0, and RODAN 1.0. Only reads alignable by Guppy were used to build each dataset, hence the N/A for unaligned reads. RODAN (nobeam) refers to a beam search of 1 which is equivalent to greedy decoding. Mismatch, deletion and insertion percentages were computed with respect to the total length of the aligned portions of all reads.

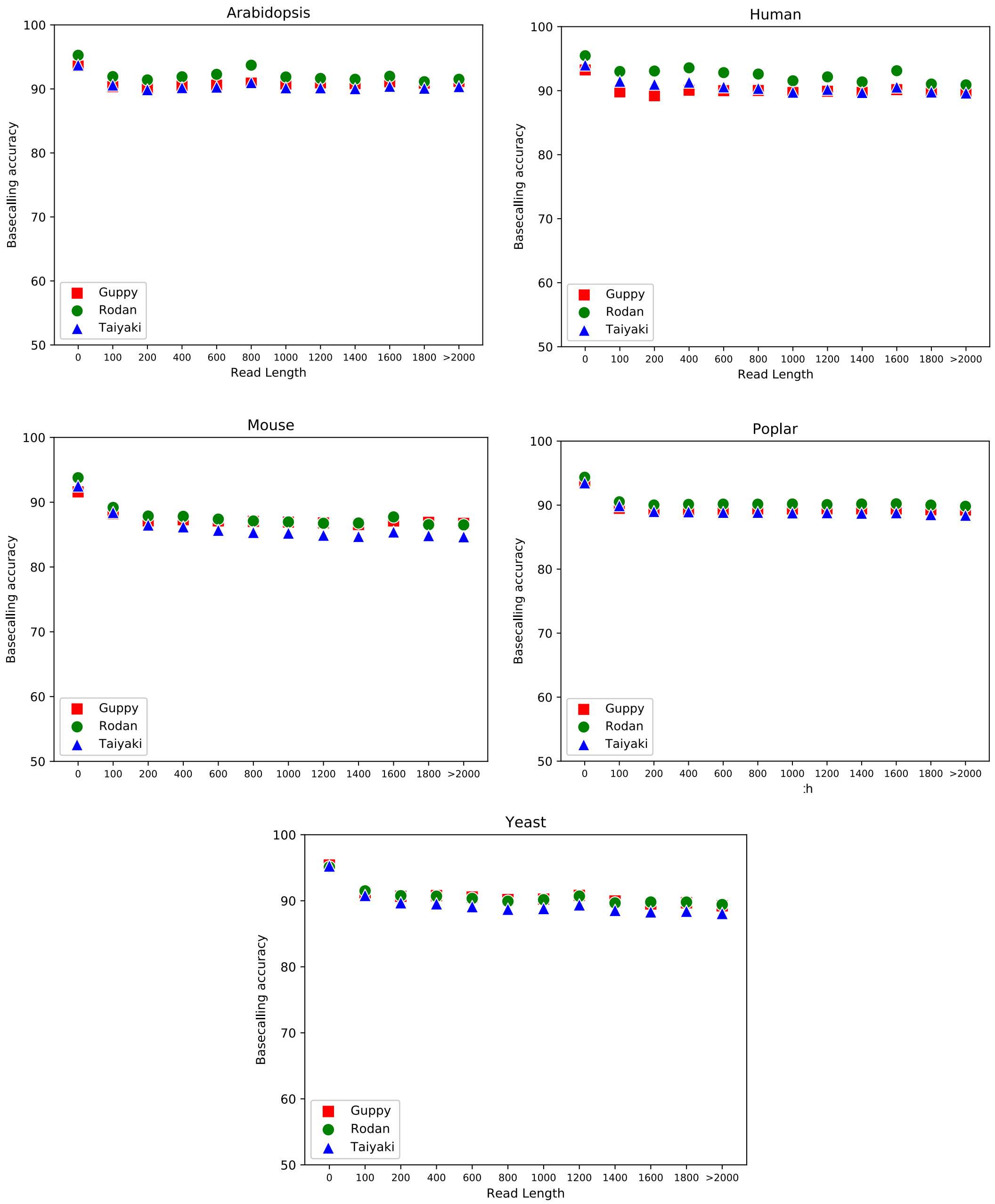

Supplementary Figure 1: Accuracy of RODAN, Guppy and Taiyaki as a function of read length across datasets.
